# Effects of audiovisual interactions on working memory: Use of the combined N-back + Go/NoGo paradigm

**DOI:** 10.3389/fpsyg.2023.1080788

**Published:** 2023-02-17

**Authors:** Yang He, Tianqi Yang, Chunyan He, Kewei Sun, Yaning Guo, Xiuchao Wang, Lifeng Bai, Ting Xue, Tao Xu, Qingjun Guo, Yang Liao, Xufeng Liu, Shengjun Wu

**Affiliations:** ^1^Department of Military Medical Psychology, Fourth Military Medical University, Xi’an, China; ^2^Department of Nursing, Fourth Military Medical University, Xi’an, China; ^3^Faculty of Humanities and Social Sciences, Aviation University of Air Force, Changchun, China; ^4^Psychology Section, Secondary Sanatorium of Air Force Healthcare Center for Special Services, Hangzhou, China; ^5^Air Force Medical Center, Air Force Medical University, Beijing, China

**Keywords:** audiovisual interaction, working memory, central executive function, cognitive load, interference effect

## Abstract

**Background:**

Approximately 94% of sensory information acquired by humans originates from the visual and auditory channels. Such information can be temporarily stored and processed in working memory, but this system has limited capacity. Working memory plays an important role in higher cognitive functions and is controlled by central executive function. Therefore, elucidating the influence of the central executive function on information processing in working memory, such as in audiovisual integration, is of great scientific and practical importance.

**Purpose:**

This study used a paradigm that combined N-back and Go/NoGo tasks, using simple Arabic numerals as stimuli, to investigate the effects of cognitive load (modulated by varying the magnitude of N) and audiovisual integration on the central executive function of working memory as well as their interaction.

**Methods:**

Sixty college students aged 17–21 years were enrolled and performed both unimodal and bimodal tasks to evaluate the central executive function of working memory. The order of the three cognitive tasks was pseudorandomized, and a Latin square design was used to account for order effects. Finally, working memory performance, i.e., reaction time and accuracy, was compared between unimodal and bimodal tasks with repeated-measures analysis of variance (ANOVA).

**Results:**

As cognitive load increased, the presence of auditory stimuli interfered with visual working memory by a moderate to large extent; similarly, as cognitive load increased, the presence of visual stimuli interfered with auditory working memory by a moderate to large effect size.

**Conclusion:**

Our study supports the theory of competing resources, i.e., that visual and auditory information interfere with each other and that the magnitude of this interference is primarily related to cognitive load.

## 1. Introduction

The human brain constantly receives a variety of information from the external environment throughout our daily lives; moreover, we usually receive information from multiple sources through multiple sensory modalities. Vision and hearing are our two main sensory modalities (Van Gerven and Guerreiro, 2016). Indeed, approximately 94% of incoming information is derived from two sensory channels, the visual and auditory channels (Feng and Yang, 2018). This information is often redundant or complementary (Leone and McCourt, 2013, 2015). Therefore, vision and hearing play an important role in higher cognitive processes (Zampini et al., 2005).

The classic studies on the effects of audiovisual interaction on higher cognitive functions include the McGurk effect and the flash illusion effect. The McGurk effect refers to the phenomenon that the visual modality can dominate simultaneously presented audiovisual information. In essence, when simultaneous but conflicting (i.e., nonmatching) audiovisual stimuli are presented, the visual stimuli interfere with the extraction of auditory information (McGurk and MacDonald, 1976), resulting in a perceptual bias in sound recognition (Gau and Noppeney, 2016). The report of the McGurk effect inspired numerous studies on audiovisual interaction. Research has indicated that extraneous sound stimuli, in addition to visual stimuli, interfere with the acquisition of auditory information and can affect visual perception. Shams et al. (2000, 2002) subsequently proposed an auditory-information-driven audiovisual interaction phenomenon. In this phenomenon, the number of (visual) flashes presented is perceived as equal to the number of (auditory) sounds presented one after another or simultaneously within 100 ms, even though these numbers are not equal. For example, when two flashes are accompanied by a sound, the flashes are incorrectly perceived as one flash; this illusion constitutes the classic sound-induced flash illusion effect (Abadi and Murphy, 2014). Based on the work by Shams et al. (2000, 2002) and Andersen et al. (2004) study showed that when flashes were simultaneously presented with an equal number of sounds (e.g., one flash with one pure tone or multiple flashes with multiple pure tones), subject accuracy in determining the number of visual stimuli increased rapidly. Notably, in the flash illusion effect, when auditory and visual stimuli appear simultaneously but the number of stimuli is inconsistent, the auditory stimuli interfere with the extraction of visual information, leading to an illusion regarding the number of visual flashes; however, this illusion is not affected by temporal or spatial variation and is relatively stable (Apthorp et al., 2013; Kostaki and Vatakis, 2016; Wang et al., 2020). This flash illusion effect represents the classic audiovisual interaction, wherein our vision affects our hearing and vice versa; whether this interaction results in interference or facilitation depends on the consistency of the simultaneously presented stimuli. For example, the presence of task-irrelevant information (e.g., pop-up ads while browsing a website, phone calls while driving) always severely interferes with the processing of task-relevant information (Forster and Lavie, 2008; Murphy et al., 2013), regardless of whether the irrelevant information is unimodal (visual or auditory stimuli) or cross-modal (audiovisual stimuli).

Working memory (WM), the core of human cognition (Zhao and Zhou, 2010), plays an important role in higher cognitive functions (Barrouillet et al., 2008; Bateman and Birney, 2019; Zhang et al., 2019), and studies have shown that the central executive (CE) serves to link WM with higher cognitive functions (Friedman and Miyake, 2017; Karr et al., 2018). Baddeley (1992, 2012) first proposed the existence of the CE in a multicomponent model of WM, arguing that the main responsibility of the CE is to control the processing of working memory; thus, the main tasks of the CE are to coordinate among the WM subcomponents, to control encoding and extraction strategies, to direct attention, and to extract information from long-term memory (Zhou and Zhao, 2010). Additionally, due to the specificity of the CE, it is considered the most central and complex component of WM (Karr et al., 2018). Furthermore, Miyake et al. (2000) divided the CE into three independent yet related functions: updating, task switching, and inhibition. Updating refers to the process of monitoring and encoding newly presented information, continuously updating information from the original memory not relevant to the current task; this function enables people to continuously update and filter task-irrelevant information and retain task-relevant information (Collette and Van der Linden, 2002). Task switching refers to attentional control, i.e., the ability to shift cognitive resources between two tasks performed simultaneously (Luo and Zhou, 2004). Inhibition refers to the ability to block access to task-irrelevant information that may be partially activated during a task, i.e., to inhibit interfering information from a highly intrusive irrelevant task when the current task competes for the same cognitive resources (Zhao and Zhou, 2011). We collectively refer to these functions of the CE as the CE functions of WM (Chen et al., 2003); these functions play an important role in higher cognitive functions.

In summary, previous studies in the literature have shown that both WM and audiovisual interactions are important for cognitive processes. First, both the modulation of the former and the integration of the latter contribute to the speed and efficiency of processing by the brain (Molholm et al., 2002). Second, electrophysiological mechanism indicators have demonstrated that both WM and audiovisual interaction are among the early steps carried out by the brain’s information-processing mechanisms (Sinnett et al., 2008; Xie et al., 2021). Third, WM involves not only the selection of different stimuli or properties from the same sense but also the selection of stimuli and information from different senses—most commonly vision and hearing (Thompson and Paivio, 1994; Xie et al., 2019). Therefore, combining these two senses in a study is bound to be of great scientific value.

However, the current task studies the CE of WM, for which the N-back task and the Go/NoGo task are the most popular paradigms (Diamond, 2013; Yeung et al., 2021). In studies of the CE of WM, the N-back task and the Go/NoGo task are the most popular paradigms (Diamond, 2013; Yeung et al., 2021). In the N-back task, participants need to judge whether the currently presented stimulus is the same as a stimulus presented N trials previously. As N increases, the cognitive load of WM (and the task demand) increases accordingly. In the Go/NoGo task, participants are instructed to react quickly to predefined “Go” stimuli and to withhold their reaction to “NoGo” stimuli. Strangely, few contemporary studies have combined the N-back and Go/NoGo paradigms to investigate the CE functions of WM in response to audiovisual interaction. Only three studies have described the interplay between the two types of tasks; these studies used a single WM task with visual or auditory information as the target task and another task that assessed only behavioral performance rather than brain activity (Guerreiro and Van Gerven, 2011; Guerreiro et al., 2013; Rienäcker et al., 2018). Among these three studies, two by Guerreiro et al. (2013) and Rienäcker et al. (2018) showed that using visual stimuli as distractors did not affect performance in the visual WM task; similarly, using auditory stimuli as distractors did not affect performance in the visual WM task. In contrast, another study by Guerreiro and Van Gerven (2011) showed that using visual stimuli as distractors affected performance in the auditory WM task but that using auditory stimuli as distractors did not affect performance in the visual WM task. This pattern contradicts the classic audiovisual interaction. The brain may need to modulate and integrate information from both vision and hearing to reach judgments about higher cognitive functions such as consciousness and behavior (Tang et al., 2016). Interestingly, the results of a recent behavioral experiment by Yang et al. that combined audiovisual interaction with a WM task suggest that audiovisual interaction may be influenced by cognitive load (He et al., 2022); specifically, under high cognitive load, there is substantial audiovisual interference, whereas under low cognitive load, there is no detectable interference or facilitation. These findings are consistent with a trade-off between speed and accuracy. Furthermore, interference effects occur when the content of the cognitive load shares similar visual characteristics with the distractor (Sinnett et al., 2007). Why are all of these variables related to visual cognitive load? While 94% of sensory information originates from both audio and visual channels, 83% comes from the visual channel and only 11% comes from the auditory channel (Xingwei et al., 2017). Therefore, visual processing is necessarily allocated more resources than auditory processing, and thus visual perception has a higher priority in sensory integration; indeed, humans exhibit visual dominance in sensory processing (Minamoto et al., 2015).

Regarding memory, the human cognitive structure consists of WM and long-term memory. WM has limited capacity, storing only 7 ± 2 items at a time (Baddeley, 2012; Shipstead et al., 2015). Previous studies have found that cognitive control of information processing influences the “processing priority”; processing priority is increased for task-relevant stimuli and decreased for task-irrelevant stimuli. Cognitive load theory suggests that excessive WM load reduces the cognitive resources available for prioritizing processing and thus prevents the suppression of distracting stimuli. Thus, under high cognitive load, distracting stimuli receive more attention and are better processed compared to conditions of low cognitive load (Lavie et al., 2004; Lavie, 2011). This theory is also supported by the finding that high WM load increases the processing of distracting stimuli (Lv et al., 2010; Holmes et al., 2014; Konstantinou et al., 2014). Thus, cognitive load may moderate audiovisual interactions, with high cognitive load potentially increasing competition for resources when visual and auditory stimuli are inconsistent. In the present study, we used a combined N-back + Go/NoGo paradigm to manipulate the cognitive load of visual WM by controlling the magnitude of N. Furthermore, we explored the effect of audiovisual interaction on the CE functions of WM and whether this effect was related to cognitive load. We proposed the following hypotheses: (1) cognitive load modulates audiovisual interactions and (2) when inconsistent auditory and visual stimuli are simultaneously presented, resource competition for processing is more pronounced, resulting in greater interference with increasing cognitive load.

## 2. Materials and methods

### 2.1. Participants

G*Power 3.1 software (Faul et al., 2009) was used to estimate the sample size. Under the premise of ensuring a medium effect size of 0.25, we set α = 0.05 and 1–β = 0.80 and calculated the minimum sample size as 44. Thus, 60 undergraduates (all male, aged 18–22 years, mean age: 19.69 ± 0.98 years) from a military medical university were publicly recruited to participate in this study. These participants were right-handed and had normal vision (and color perception), hearing, and intelligence (according to college entrance examination scores) and had no history of mental or neurological diseases. The average college entrance examination score was 600.25 ± 24.89 points. All subjects performed both a unimodal and a bimodal WM task within a session to assess CE functions. The order of the three cognitive tasks was pseudorandomized among all participants, and a Latin square design was used to account for order effects. All participants volunteered to participate in the experiment and provided written informed consent. They also received monetary compensation for their participation. Finally, this study was conducted in accordance with the Declaration of Helsinki and was approved by the Ethical Committee for Drug Clinical Trials of the First Affiliated Hospital of the Fourth Military Medical University (KY20224106-1).

### 2.2. Experimental design

The present study used an experimental design with three conditions: a unimodal visual N-back task, where only digits were memorized; a unimodal auditory Go/NoGo task, where only judgments of sound stimuli were needed; and finally, a combined bimodal visual N-back + auditory Go/NoGo task, where both sounds and digits were present. The unimodal and bimodal tasks were compared using paired-sample t-tests.

### 2.3. Materials and tasks

#### 2.3.1. Stimuli

All tasks were compiled in and presented with E-Prime 3.0. The visual stimuli (Arabic numerals 0–9) were presented on a Lenovo computer monitor with a resolution of 1,024 × 768 pixels, and auditory stimuli were presented through headsets with an amplitude of 70 dB. The first auditory stimulus was a low-pitched “toot” (262 Hz), and the second auditory stimulus was a high-pitched “beep” (524 Hz). The subjects were seated 60–80 cm away from the computer screen. Before the start of the experiment, the subjects were first asked to put on the earphones and then were presented with instructions and asked to read them carefully. After participants read the instructions, the content of the experimental task was introduced; experimenters were available to answer participant questions. In all tasks, a white fixation cross was displayed in the center of the screen for 500 ms at the start, followed by presentation of the test stimulus for 800 ms. Subsequently, subjects indicated their decision by pressing a button, and the next stimulus was presented after the button press or 3,000 ms, whichever was shorter.

#### 2.3.2. Unimodal visual N-back task

**Figure 1:**
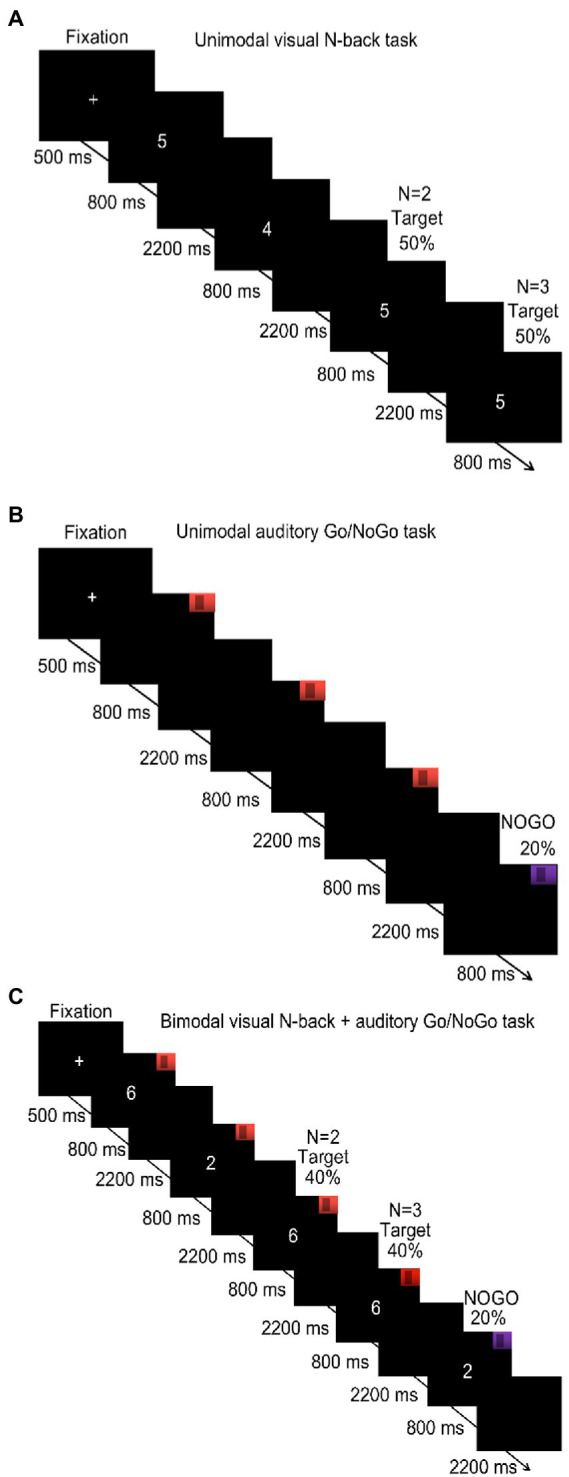
Procedure of the unimodal and bimodal tasks under low and high cognitive loads (N of 2 and 3, respectively). **(A)** Unimodal visual N-back task under low (*N* = 2) and high cognitive load (*N* = 3); **(B)** unimodal auditory Go/NoGo task; **(C)** bimodal visual N-back + auditory Go/NoGo task under low (*N* = 2) and high cognitive load (*N* = 3).

The N-back task was used to evaluate the updating of visual WM. Previous studies have found that the 1-back condition is a pure memory retention task that mainly measures the short-term memory of subjects, while the 3-back condition represents the maximum memory load under which subjects can respond correctly (Harvey et al., 2005; Román et al., 2015). Therefore, we chose *N* = 2 for the low cognitive load and *N* = 3 for the high cognitive load in the WM tasks. In this N-back task, a series of Arabic numerals (0–9; e.g., 2, 4, 2, and 1) was presented, and the participants decided if the current stimulus was identical to the stimulus two trials earlier (*N* = 2 condition) or three trials earlier (*N* = 3 condition). Targets were presented in 50% of the trials. If stimuli were identical, the participants were instructed to press “F” on the keyboard with their left hand; if the stimuli were not identical, they were instructed to press “J” with their right hand. The experimental procedure is shown in Figure 1A. The key assignments were the same for all participants, and they were asked to respond quickly and accurately. There was only one block in the 2-back condition. This block consisted of 40 trials over 3 min. Before the block, a training session of 20 trials (1 min) was administered with feedback provided for correct or incorrect responses. When accuracy in this training stage reached 80%, the participants were considered to understand the task and entered the formal experiment. Otherwise, the participants repeated the training session. The procedure of the 3-back condition was the same as the 2-back condition. The participants completed the 2-back condition before the 3-back condition. The final outcomes were the reaction times and accuracy of participants in each condition.

#### 2.3.3. Unimodal auditory Go/NoGo task

The Go/NoGo task was used to evaluate the inhibition of auditory WM (Diamond, 2013). In this task, the subjects were randomly presented with one of two sound stimuli. When they heard the Go stimulus (524 Hz), the subjects were asked to press the space key. When they heard the NoGo stimulus (262 Hz), subjects were instructed not to respond; NoGo trials constituted 20% of the experiment. The experimental procedure is shown in Figure 1B. The task consisted of a single block, approximately 4 min in duration, that included a total of 50 trials. Before the formal task started, a 1-min (20-trial) practice session was administered, with feedback provided for correct or incorrect responses. When accuracy in this training stage reached 80%, the participants were considered to understand the task and entered the formal experiment. Otherwise, the participants repeated the training session. The final outcomes were the reaction times of subjects on the Go trials and the accuracy rates on the NoGo trials.

#### 2.3.4. Bimodal visual N-back + auditory Go/NoGo task

A combined N-back + Go/NoGo bimodal task was used to evaluate the updating and inhibition of WM under conditions of audiovisual interaction. In this task, the participants were simultaneously presented with a sound stimulus and a visually presented number, both of which were randomly selected. The participants were asked not only to judge the sound that they heard but also to remember the number presented. Upon hearing the 424-Hz sound, the participants were asked to judge whether the current stimulus was identical to the stimulus presented two trials earlier (*N* = 2; low cognitive load) or three trials earlier (*N* = 3; high cognitive load). Of the total trials, 40% were target trials. If the stimuli were identical, participants were instructed to press “F” on the keyboard with their left hand; if the stimuli were not identical, participants were instructed to press “J” with their right hand. However, upon hearing the 262-Hz sound, participants were instructed to withhold their response, regardless of whether the current stimulus was the same or different from the stimulus presented two (*N* = 2 condition) or three trials earlier (*N* = 3 condition). Of the total trials, 20% involved withholding a response. The assigned keys were identical for all participants, and they were instructed to respond quickly and accurately. There was only one block in the visual 2-back + auditory Go/NoGo condition, which consisted of one run that lasted 4 min (50 trials). The visual 2-back + auditory Go/NoGo condition started with a training run of 1 min (20 trials) with feedback indicating correct or incorrect reactions. When the accuracy rate in the training stage reached 80%, the participants were considered to have an appropriate understanding of the task, and the formal experimental stage was initiated. Otherwise, the participants repeated the training. The visual 3-back + auditory Go/NoGo condition was the same as the visual 2-back + auditory Go/NoGo condition. The participants completed the visual 2-back + auditory Go/NoGo condition first and then the visual 3-back + auditory Go/NoGo condition. The program illustration is shown in Figure 1C. The bimodal visual N-back + auditory Go/NoGo task provided an equal quantity of target trials to the unimodal N-back task and the unimodal Go/NoGo task to facilitate subsequent comparisons. The final result was to record the reaction time and accuracy of the subjects in the N-back task and Go/NoGo task.

### 2.4. Statistical analysis

SPSS 25.0 software was used to analyze the behavioral data. First, the data were screened to identify reaction times >3 standard deviations from the mean; these data were then excluded from the analysis. Second, the normality of the accuracy and reaction time data was analyzed on the unimodal (visual or auditory) and bimodal (audiovisual) tasks; both the accuracy and reaction time were normally distributed. Therefore, repeated-measures analysis of variance (ANOVA) was used to examine both accuracy and reaction time in the (unimodal and bimodal) N-back task. One-way ANOVA was used to assess both accuracy and reaction time in the (unimodal and bimodal) Go/NoGo task. Finally, the effect size η^2^ was used to evaluate the effect of audiovisual interaction on the CE functions of WM. The effect size was considered small when η^2^ = 0.01, medium when η^2^ = 0.06, and large when η^2^ = 0.14 (Chaolu, 2003).

## 3. Results

### 3.1. Comparisons between the unimodal visual N-back task and bimodal visual N-back + auditory Go/NoGo task

The accuracy rates and reaction times on the unimodal N-back task (visual) and bimodal N-back task (audiovisual interaction) under low and high cognitive load were analyzed and are shown in Table 1.

**Table 1:** Behavioral performance on the unimodal N-back task (visual) and bimodal N-back task (audiovisual interaction; mean ± standard deviation).

Cognitive load	Task type	Behavioral results	
		Accuracy	Reaction time (ms)
Low (*N* = 2)	Unimodal N-back (visual)	0.94 ± 0.05	922.93 ± 179.22
	Bimodal N-back (audiovisual interaction)	0.92 ± 0.05	1015.52 ± 277.54
High (*N* = 3)	Unimodal N-back (visual)	0.89 ± 0.08	983.23 ± 292.35
	Bimodal N-back (audiovisual interaction)	0.83 ± 0.08	1208.01 ± 240.77

**Figure 2:**
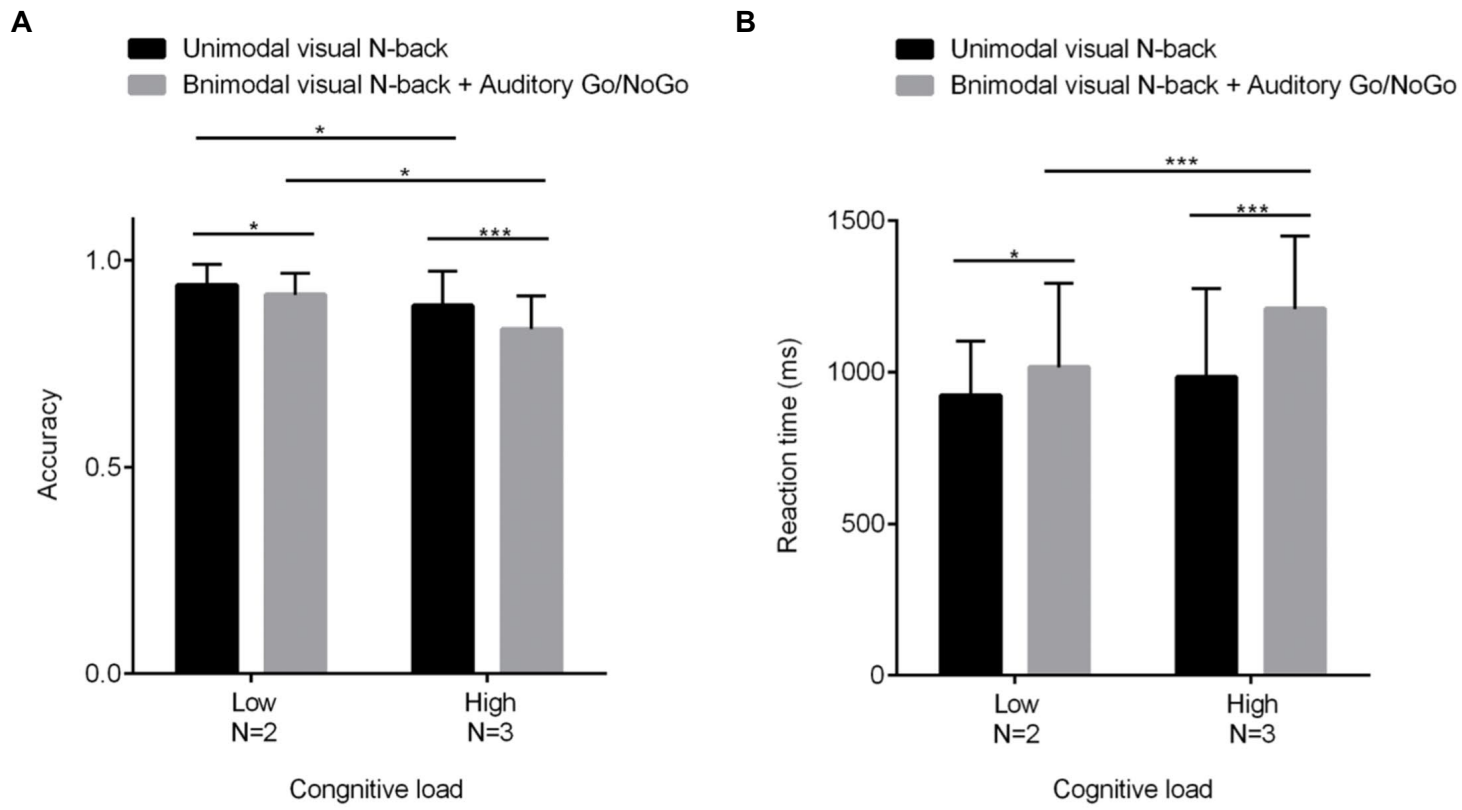
Behavioral performance (mean scores and standard deviations) in terms of accuracy **(A)** and reaction times **(B)** on unimodal and bimodal visual tasks with auditory distractors under different visual cognitive loads. Among them, *N* = 2 represents low cognitive load, and *N* = 3 represents high cognitive load. As the visual cognitive load increased, the auditory interference became more pronounced, with greater differences in terms of both reaction time and accuracy. All the data are presented as the mean ± S.D. (*n* = 60); *p < 0.05, **p < 0.01, ***p < 0.001.

2 (cognitive load: low load N = 2, high load N = 3) × 2 (modality type: unimodal visual, bimodal audiovisual) repeated-measures analysis of variance (ANOVA) revealed a significant main effect of cognitive load [*F*(1, 60) = 62.617, *p* < 0.001, η^2^ = 0.515], with a significantly lower correct rate on the N-back task in the high-load condition than in the low-load condition[*N* = 2: *p* < 0.05, η^2^ = 0.102; *N* = 3: *p* < 0.001, η^2^ = 0.209]; a significant main effect of modality type [*F*(1, 60) = 23.272, *p* < 0.001, η^2^ = 0.283], with a lower correct rate on the N-back task in the bimodal (audiovisual interaction) than in the unimodal (visual) condition; and a marginal interaction between the two [*F*(1, 60) = 3.715, *p* = 0.059, η^2^ = 0.069]. Since the effect size was large enough to be considered moderate, further simple-effect tests were conducted, as shown in Figure 2A; these tests revealed a significantly lower correct rate on the bimodal (audiovisual interaction) N-back task than on the unimodal (visual) N-back task in both high-and low-load conditions.

In terms of reaction times, the main effect of cognitive load was significant [*F*(1, 60) = 14.167, *p* < 0.001, η^2^ = 0.194], with reaction time in the N-back task being significantly greater in the high-load condition than in the low-load condition[*N* = 2: *p* < 0.05, η^2^ = 0.080; *N* = 3: *p* < 0.001, η^2^ = 0.287]; the main effect of modality was significant as well [*F*(1, 60) = 23.449, *p* < 0.001, η^2^ = 0.284], with reaction time on the N-back task being greater in the bimodal (audiovisual interaction) than in the unimodal (visual) condition; the cognitive load × modality type interaction was significant [*F*(1, 60) = 3.715, *p* < 0.05, η^2^ = 0.082], and further simple-effect tests showed that in each cognitive load condition, the modalities were significantly different (*ps* < 0.05) as shown in Figure 2B.

### 3.2. Comparisons between the unimodal auditory Go/NoGo task and bimodal auditory Go/NoGo + visual N-back task

The accuracy and reaction times on the unimodal Go/NoGo task (auditory) and bimodal Go/NoGo task (audiovisual interaction) under different cognitive loads were analyzed and are shown in Table 2.

**Table 2:** Behavioral performance on the unimodal Go/NoGo task (auditory) and bimodal Go/NoGo task (audiovisual interaction; mean ± standard deviation).

Cognitive load	Task type	Behavioral results	
		Accuracy	Reaction time (ms)
	Unimodal Go/NoGo (auditory)	0.97 ± 0.04	510.08 ± 139.63
Low (*N* = 2)	Bimodal Go/NoGo (audiovisual interaction)	0.96 ± 0.05	606.45 ± 199.05
High (*N* = 3)	Bimodal Go/NoGo (audiovisual interaction)	0.94 ± 0.06	709.59 ± 305.94

**Figure 3:**
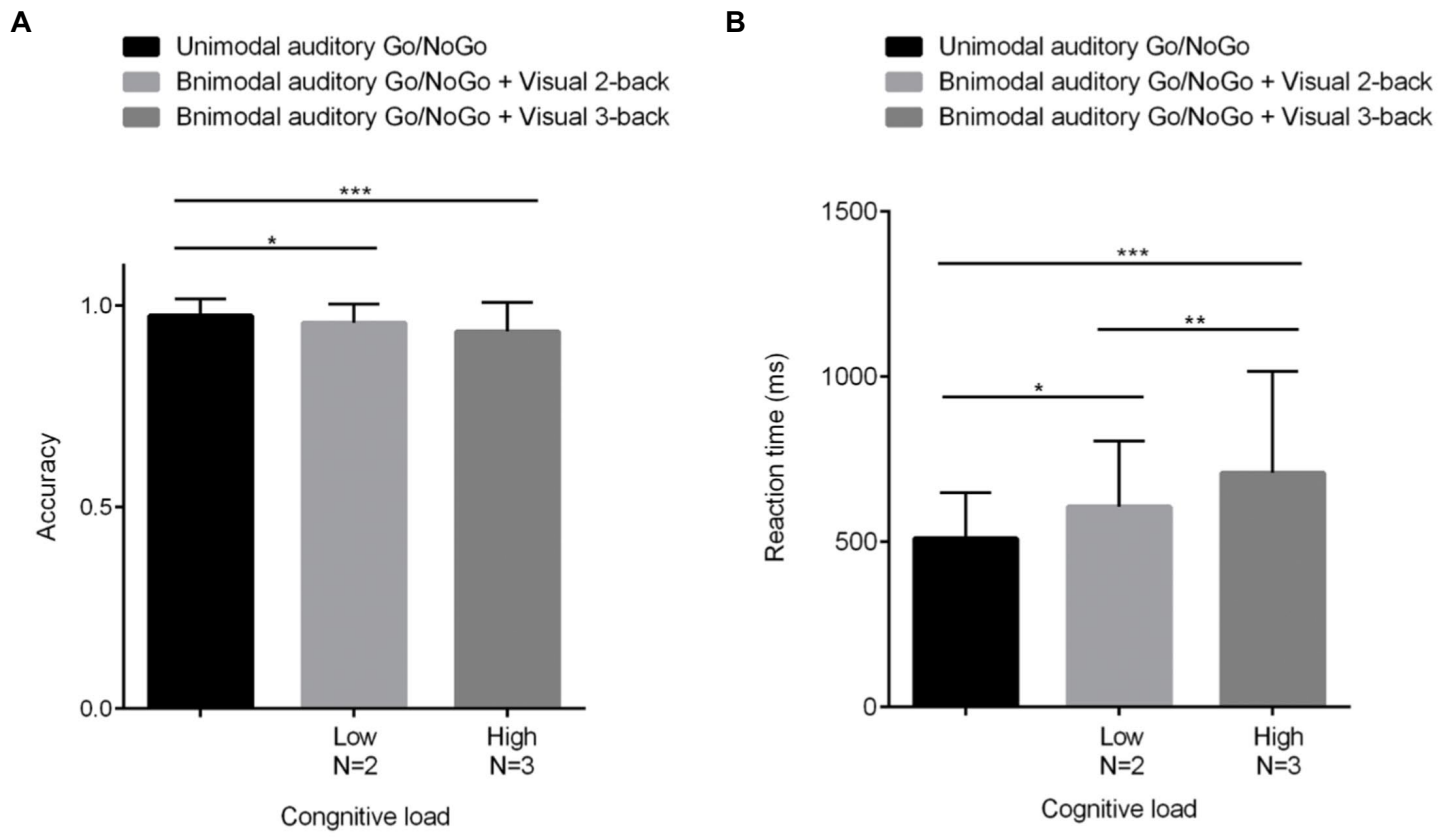
Behavioral performance (mean scores and standard deviations) in terms of accuracy **(A)** and reaction times **(B)** on the unimodal and bimodal auditory tasks with visual distractors under different visual cognitive loads. Among them, *N* = 2 represents low cognitive load, and *N* = 3 represents high cognitive load. As the visual cognitive load increased, the differences in reaction time and accuracy of WM became greater in both the unimodal and bimodal tasks. All the data are presented as the mean ± S.D. (*n* = 60); *p < 0.05, **p < 0.01, ***p < 0.001.

The accuracy on NoGo trials (hereafter, NoGo accuracy) under different visual cognitive loads is shown in Figure 3A and was analyzed by one-factor repeated-measures analysis of variance (ANOVA). Mauchly’s test of sphericity yielded W = 0.836, *p* < 0.01. Therefore, the Greenhouse–Geisser method was used to correct the degrees of freedom. The main effect of visual cognitive loads was significant [*F*(1.718, 60) = 8.746, *p* < 0.01, η^2^ = 0.129]. Further *post hoc* comparative analysis indicated that accuracy for NoGo trials in the unimodal auditory Go/NoGo task was higher than in the bimodal auditory Go/NoGo + visual 2-back condition (N = 2: *p* < 0.05, η^2^ = 0.094). With an increasing cognitive load, NoGo trials of accuracy for bimodal auditory Go/NoGo + visual 3-back was significantly lower than for unimodal auditory Go/NoGo trials (N = 3, *p* < 0.001, η^2^ = 0.196). However, there was no difference between the accuracy rate of NoGo trials in bimodal auditory Go/NoGo + visual 3-back task and the accuracy rate of NoGo trials in bimodal auditory Go/NoGo + visual 2-back task (*p* > 0.05).

The reaction times on Go trials (hereafter, Go reaction times) under different visual cognitive loads are shown in Figure 3B and were analyzed using a one-factor repeated-measures analysis of variance (ANOVA). Mauchly’s test of sphericity yielded W = 0.710, *p* < 0.001. Therefore, the Greenhouse–Geisser method was used to correct the degrees of freedom. The results indicated that the main effect of visual cognitive loads was significant [*F*(1.551, 60) = 13.303, *p* < 0.001, η^2^ = 0.184]; the Go reaction times on the bimodal auditory Go/NoGo + visual N-back task were longer than those on the unimodal auditory Go/NoGo task. Further *post hoc* comparative analysis indicated that reaction times for GO trials in the unimodal auditory Go/NoGo task were lower in the low-load condition than in the bimodal auditory Go/NoGo + visual 2-back condition (*N* = 2: *p* < 0.05, η^2^ = 0.121). With increasing cognitive load, reaction times for bimodal auditory Go/NoGo + visual 3-back unimodal Go trials were significantly longer than those for the auditory Go/NoGo task (*N* = 3: *p* < 0.001, η^2^ = 0.227). The longest response time was observed for bimodal auditory Go/NoGo + visual 3-back Go trials, which were significantly different from bimodal auditory Go/NoGo + visual 2-back Go trials (*p* < 0.01). A correlation was computed between accuracy and RT to determine whether there were speed-accuracy trade-offs. There were significant correlations [*N* = 3: *r*(60) = −0.066, *p* < 0.05].

## 4. Discussion

The N-back task and the Go/NoGo task are among the most widely used tasks to study the CE functions of WM (Diamond, 2013; Yeung et al., 2021). The present study used a combined N-back + Go/NoGo paradigm to manipulate cognitive load by controlling the magnitude of N and to explore the effect of audiovisual interaction on the CE functions of WM to determine whether this effect was related to cognitive load. Thus, we aimed to provide a theoretical basis for the effect of audiovisual interaction on information processing in higher cognitive functions. The N-back task was mainly used to evaluate updating (a CE function of WM) with unimodal (visual) tasks; the Go/NoGo task was used to evaluate inhibition (a CE function of WM) with unimodal (auditory) tasks.

### 4.1. Differences in the central executive function of visual working memory in unimodal and bimodal conditions under different cognitive loads

On the auditory Go/NoGo task under low visual cognitive load, participants differed in both accuracy and reaction time on the N-back task between the bimodal and unimodal conditions. That is, compared with the CE functions of WM in the unimodal (visual) condition, the CE functions of WM under the bimodal condition (i.e., audiovisual interaction) decreased in terms of updating, as indicated by lower accuracy rates and longer reaction times. This pattern corresponds to the classic flash illusion effect and the theory of resource competition, in which simultaneously presented auditory and visual stimuli with inconsistent content result in audiovisual interference, i.e., the presence of auditory stimuli interferes with the extraction of visual information (Meylan and Murray, 2007; Dehais et al., 2019; Marsh et al., 2020). Our findings suggest that the presence of auditory distractors produced a moderate interference with visual WM in terms of effect size (Chaolu, 2003). This result is due to the limited WM capacity; cognitive load is negatively correlated with cognitive resource reserve when cognitive resources are effectively allocated and controlled (Horat et al., 2016). In other words, the lower the cognitive load perceived by the individual, the more adequate the cognitive resource reserve, and therefore more effective inhibition of irrelevant stimuli can be achieved. Thus, the presence of auditory distractors produced only moderate interference with visual WM. However, under high cognitive load, the presence of auditory distractors produced large interference with visual WM in terms of effect size (Chaolu, 2003). This result is due to the occupation of more cognitive resources used to direct attention under high cognitive load, preventing effective inhibition of interfering stimuli; thus, individuals are more susceptible to interference from task-irrelevant stimuli. In other words, irrelevant stimuli are better processed under high cognitive load (Lavie and Tsal, 1994; Ahmed and de Fockert, 2012; Horat et al., 2016). Indeed, the CE functions of visual WM in the bimodal condition (i.e., audiovisual interaction) had a significantly lower accuracy rate and longer reaction times compared to the CE functions of visual WM in the unimodal condition; therefore, there was no speed-accuracy trade-off. Compared to consistent bimodal (i.e., audiovisual) stimuli, our brains have difficulty processing inconsistent bimodal stimuli, which to impairs reaction times (Misselhorn et al., 2019), resulting in lower accuracy rates, longer reaction times, and the absence of a speed-accuracy trade-off (Thelen et al., 2012; Heikkilä et al., 2015; Bigelow and Poremba, 2016). Furthermore, one of the biggest differences between auditory and visual distractors is that auditory distractors can only be processed under high cognitive load, which triggers interference effects (Tellinghuisen and Nowak, 2003). Therefore, under high visual cognitive load, our auditory distractors were better processed (Alderson et al., 2017). Moreover, the cognitive load condition in the task affects both the accuracy of WM (Luck and Vogel, 1997) and the interference effect of extraneous distractors (Zhang et al., 2011). For example, the attentional load theory proposed by Lavie et al. (2004) suggests that interference effects increase with cognitive load, i.e., under low cognitive load, although both target and distractor stimuli are perceptible, goal-oriented attentional control can suppress interference effects, whereas under high cognitive load, it is difficult to suppress top-down processing because of the lack of cognitive resources. Therefore, auditory distractors under high cognitive load produced large interference compared to under low cognitive load, consistent with our hypothesis.

### 4.2. Differences in the central executive function of auditory working memory in unimodal and bimodal conditions under different cognitive loads

On the visual N-back task under low cognitive load, we found that the CE functions of WM in the unimodal (auditory) condition were higher than those in the bimodal (audiovisual) condition, as mainly indicated by decreased accuracy and longer reaction times. This pattern corresponds to the classic McGurk effect, as simultaneous yet inconsistent audiovisual stimuli lead to competition for limited WM resources, i.e., the presence of visual stimuli interferes with the extraction of auditory information (Spence et al., 2012; Sandhu and Dyson, 2016; Sörqvist et al., 2016), which, in turn, affects processing priority (Evans and Treisman, 2010). In addition, we found that under higher visual cognitive load, inhibition (a CE function of auditory WM) in the bimodal (audiovisual) condition was consistently lower than that in the unimodal (auditory) condition, mainly reflected in the significantly lower accuracy rate and significantly longer reaction time. This difference arises because the identification of auditory (target) stimuli is related to visual cognitive load; as the visual cognitive load increases, the correct identification rate of auditory stimuli will significantly decrease (Dehais et al., 2019), resulting in longer reaction times and significantly lower accuracy in the presence of distracting stimuli (Weil et al., 2012; Murphy et al., 2016). Moreover, previous studies that used auditory stimuli as targets and visual stimuli as distractors to assess audiovisual interactions found that interference affects brain responses to a certain extent (Evans and Treisman, 2010). The magnitude of interference depends on the visual cognitive load, i.e., as the visual cognitive load increases, the interference effect becomes more pronounced (Sadeh and Bredemeier, 2011). This pattern is consistent with our hypothesis that in the presence of visual distractors under high visual cognitive load, there will be a large interference effect on both reaction times and accuracy compared to that under low cognitive load (Chaolu, 2003). This is consistent with recent findings from researchers on spatial conflict tasks under audiovisual interactions. Compared to positionally congruent audiovisual stimuli, the recognition of auditory target stimuli under positionally incongruent audiovisual stimulus interactions is related to our visual cognitive processing load, and the correct rate of recognition of auditory target stimuli will significantly decrease as the visual cognitive processing task becomes increasingly complex (Dehais et al., 2019). This recent result may provide valid evidential support for our experimental findings that audiovisual interaction interferes with working memory task performance when the processing content of visual and auditory stimuli is inconsistent, i.e., it is manifested by a decrease in the correct rate of inhibition and a prolonged response time, following the theory of resource competition under audiovisual interaction, and the magnitude of this resource competition depends mainly on cognitive load (Zhu et al., 2022). As the visual interference condition increased from 2-back to 3-back, the correct rate of the inhibition function of working memory under audiovisual interaction was the lowest, the reaction time was the longest, and the effect size increased from a medium effect to a large equal effect size.

## 5. Limitations

The present study has three limitations. First, regarding the experimental design, we mainly considered the predominance of visual information in audiovisual interactions and manipulated visual cognitive load; we did not investigate the potential predominance of auditory information in these interactions. Future studies should utilize appropriate designs (i.e., manipulate auditory cognitive load) to improve the reliability of findings and observe whether the same pattern of results is obtained. Second, further investigation is needed regarding whether the results in the bimodal task are due to the combination of tasks or the interaction between senses; therefore, we must refine the experimental design in future studies. For example, we plan to run a unimodal visual Go/NoGo task and a unimodal auditory N-back task in order to assess exactly what causes the interesting experimental results in the bimodal visual N-back + auditory Go/NoGo task. Finally, this study used only behavioral outcomes to explore whether the occurrence of audiovisual interactions was related to cognitive load; we thus lacked the support of electrophysiological measurements. Future studies should employ advanced electrophysiological instrumentation, such as electroencephalography (EEG), functional near-infrared spectroscopy (FNIRS), and functional magnetic resonance imaging (fMRI) techniques, to explore whether the occurrence of audiovisual interaction differs in specific event-related potential (ERP) components or brain regions(Li et al., 2019; Xi et al., 2020a,b).

## 6. Conclusion

Our findings support the competition theory of resources, i.e., vision and hearing interfere with each other, and the magnitude of this interference is related to cognitive load. Specifically, when visual and auditory stimuli are inconsistent, the effect of audiovisual interaction on the CE functions of WM follows this theory. Thus, under high cognitive load, resource competition is more pronounced, and greater interference is observed in bimodal (i.e., audiovisual) tasks than in unimodal (auditory or visual) tasks.

## Data availability statement

The original contributions presented in the study are included in the article/supplementary material, further inquiries can be directed to the corresponding author/s.

## Ethics statement

The studies involving human participants were reviewed and approved by KY20224106-1. The patients/participants provided their written informed consent to participate in this study.

## Author contributions

YH, TY, and CH proposed the experiment, designed the procedure, and wrote the manuscript. SW and XL provided useful comments on the experiment and helped to revise the manuscript several times. KS, YG, XW, LB, TiX, TaX, QG, and YL assisted with participant recruitment and administered the experiment. All authors contributed to the article and approved the submitted version.

## Funding

This study was supported with grants from the Key project of PLA Logistics Research Program during the 14th Five-Year Plan period (Grant BKJ21J013), the Air Force Medical University of Aviation Medicine Major Problems of Scientific Research (Grant 2019ZTD04), the Key Project of the PLA Air Force Equipment and Military Scientific Research Program (Grant KJ20182A030138), and the Military Science and Technology Commission Basic Strengthening Plan Project (2020-JCJQ-ZD-263-04).

## Conflict of interest

The authors declare that the research was conducted in the absence of any commercial or financial relationships that could be construed as a potential conflict of interest.

## Publisher’s note

All claims expressed in this article are solely those of the authors and do not necessarily represent those of their affiliated organizations, or those of the publisher, the editors and the reviewers. Any product that may be evaluated in this article, or claim that may be made by its manufacturer, is not guaranteed or endorsed by the publisher.

## Abbreviations

WM: Working memory
CE: The central executive
