# Supplementary figures and images for: Effects of audiovisual interactions on working memory: Use of the combined N-back + Go/NoGo paradigm
Source: Front Psychol. 2023 Feb 17;14:1080788. doi: 10.3389/fpsyg.2023.1080788 (PMC9982107; doi:10.3389/fpsyg.2023.1080788)

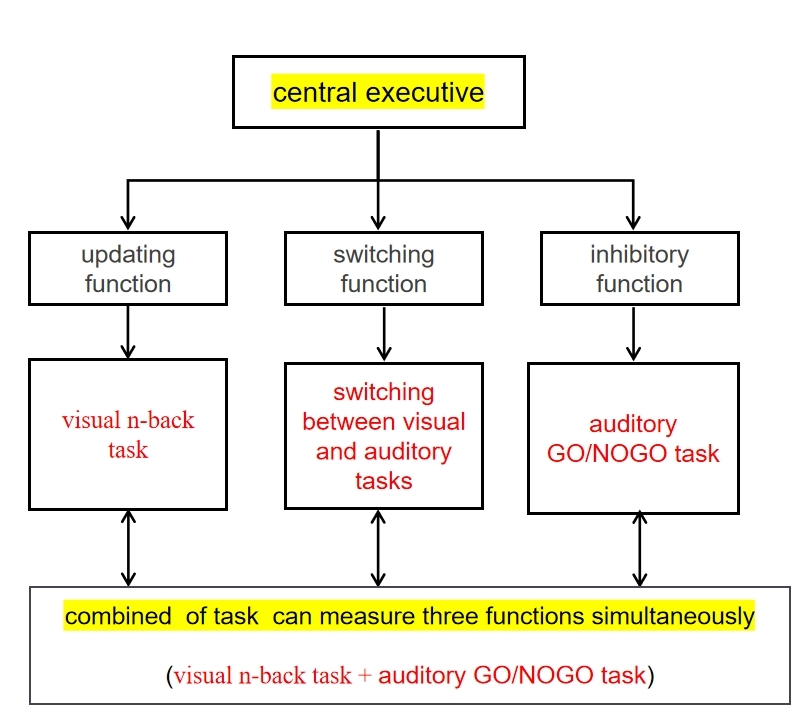

Supplement: Supplementary file 1 [file Image_1.JPEG]
